# Medical Items and News of Interest to the Physician

**Published:** 1875-04

**Authors:** 


### For the Use of Physicians.

CULLED FROM THE STANDARD MEDICAL JOURNALS OF THE WORLD.

NOTE.—These formulæ are intended only for the regular practitioner of medicine, and should be employed only by him, or under his immediate supervision.

**Bedsores.**

Sir James Paget says the best wash for hardening the skin, to prevent bedsores, is one part of sweet spirits of nitre to three parts of water.

**Enlargement of the Spleen.**

For this frequent sequela of intermittent, Dr. Bernard has found much benefit from bromide of potassium, fifteen to thirty grains daily, continued several weeks.

**Quinine in Epistaxis.**

A writer in the *Lancet* says quinine is the remedy in epistaxis. He says he has tried it more than twenty times, often in aged people, and never has found it to fail.

**Coryza.**

According to French authority, powdered camphor, sprinkled with tincture of iodine, and inhaled by the nostrils, constitutes one of the most prompt and certain remedies for coryza, or "cold in the head."

**Chilblains and Frosted Feet.**

Remedies for these troubles are in order at this season. The most recent is cajeput oil. A writer says it is the most efficacious. Apply it locally morning and evening. Often one application is sufficient for light cases.

**Dropsy of the Joints.**

Dr. Bergeret recommends for this the continued application of hot sand. He first envelopes the joint in cotton wool, and then surrounds it with small bags of very hot sand, putting a blanket over them to retain the heat, and renewing them as needed. The dropsy should disappear in a few days.

**Iodide of Potassium in Diphtheria.**

A writer in the *British Medical Journal* says: In diphtheria, iodide of potassium is looked upon by many practitioners as the best remedy we possess. Here its alterative and relative actions are laid aside, and we have it doing duty as an antidote to the dipheritic poison; although, so far as can be seen, it exercises no new influence.

**Asthma.**

The hypodermic injection of chloral, dissolved in twenty minims of water, will relieve in ten minutes a violent asthmatic spasm, according to Surgeon Major Baille, R. N.

**Ozæna.**

Dr. Eyselein, of Blankenburg, reports a very obstinate case of ozæna, which he had cured by painting the ulcerating nasal membrane with a solution of chlorate of potash, one part to six of water, by weight.

**For Painful Hemorrhoids.**

℞.
Ext. hyoscyam.,
Pulv. saffron, āā, ʒ ijss.
Plumbi acetat, ℥ i.
Glycerole of starch, ℥ i. M.

**In Secondary Syphilis.**

℞.
Hydrarg. chloridi corr., grs. ij.
Chlor. ammonii, grs. 120.
Tinct. ferri. chlor., grs. 180.
Syrup, f. ℥ j.
Aquæ cinnamomi q. s. *ad.* ℥ iv. M.
Sig. Teaspoonful in water thrice daily.

**Management of Hydrocele.**

Mr. E. Lund, of the Manchester Hospital, in a recent report, says: "The secret of success in the management of hydrocele consists in thoroughly distending the sac of the hydrocele, by first noting the number of fluid ounces of serum drawn off, and then ejecting at least one ounce more of port wine, so as thoroughly to expand and fill the sac."

**Almost a Specific in Diphtheria.**

℞.

Quinæ sulph, grs. xvj.

Liq. ammon acet, ℥ iss.

Acid acetici dil, ʒ i.

Aquæ destill, ʒ iv.

Syrupi, ʒ iv.

Tinct. ferri chl., ʒ iij. M.

Teaspoonful every four hours.

**Pityriasis Capitis.**

For this often obstinate complaint cocoa butter, castor oil, almond oil, of each five drachms; turbith mineral, fifteen grains. Smear the scalp with the ointment thrice daily, and wash the head with soap three times each week. It is necessary to cut the hair short and brush the head briskly, so as to remove the pellicles and lay bare the parasite. A larger quantity of turbith mineral may be used without inconvenience.

**For Chronic Hepatitis.**

℞.

Resinæ podophylli, grs. iv.

Extract leptandriæ, grs. xlviij.

Irisin, grs. xvi.

M. Div. in pil. No. xvj. Sig. One every other night.

**Dyspepsia Pills.**

℞.

Zinci Sulph., grs. iv.
Ext. nucis vomicæ, grs. iv.
Podophyllin, grs. ij.
Ext. rhei., grs. xxiv.

M. Div. in pil. No. xvj. Sig. One pill thrice a day.

**Recurrent Chronic Bronchitis.**

Many persons are subject to attacks of chronic winter cough, commencing with difficult breathing and a hard dry cough. In this stage, says Dr. Lawrie, of Glasgow, no remedy gives such prompt relief as iodide of potassium. It promotes and restores the bronchial secretion, and tranquilizes the respiration. When free secretion has set in, it should be abandoned.

**Catarrh and Paralysis of Bladder.**

Dr. Willimsky of Griefswald, in an inaugural theses, recommends the watery solution of ergot. A dilute solution should be thrown into the bladder, in paralysis, thrice a day, in acute catarrh of the organ, once a day. In either case the bladder must be thoroughly emptied, before the ergot is used. Successful cases are quoted in support of this treatment.

**Itch.**

Dr. F. W. Clemens reports, in the *Allgemeine Medicinische Centralzeitung*, excellent results, with the following ointment:

℞.

Arsenious acid, grs. ij.
Carbonate of potash, grs. x.
Spirit of soap, ʒ j.
Water, to ℥j. M.

Sig. Rub twice daily on the affected parts.

**A Diagnostic Hint in Old Ulcers.**

In a lecture on chronic ulcers of the leg, in the Detroit *Review of Medicine and Pharmacy*, Dr. McGraw says: I will give you a rule for diagnosis. It has very few exceptions. *Idiopathic ulcers, which occur in the upper third of the leg, are syphilitic.* When you are called upon to treat such cases, inquire whether the ulcer originated in an injury; if not, treat the patient for syphilis, without further ado.

**Cough Mixture.**

℞.

Mist. glycyrrh. comp., ℥ iv.

Syrup pruni. virg., ℥ ii.

Syrup senegæ, ʒ v.

Chloridi ammonii, ʒ ii.

Morphiæ sulph., grs. ii.

Syrup accaciæ, q.s. *ad.*, ℥ viij. M.

Sig: Dessertspoonful every three hours.

**Treatment of Biliary Calculi.**

Dr. Laborde, of Paris, states that in this terribly painful affection morphia and hydrate of chloral, administered simultaneously, form the best mode of treatment. They exert both an anasthetic and paralyzing influence, with the effect of producing cessation of the spasmodic state, distention of the passages, and accumulation of the biliary fluid, which act on the foreign body by a kind of *vis a tergo*, and force it down the intestines.

**Rheumatism.**

Dr. Copeman, in the *British Medical Journal*, recommends a tincture of the leaves of the artichoke, *cynara*, gathered while they are full of juice, just before the top of the vegetable is fit for food. In his experience no medicine has been so efficacious, both in acute and chronic rheumatism. His formula is as follows:

℞.

Potassa bicarb., ʒ j.
Aquæ camph. ad., ℥ viij.
Tincture cynaræ, ℥ j.
Syrup papaveris alb., ℥ ss. M.

Sig: Take two tablespoonfuls every four hours, with as much of the extract of cynara as would make two moderately sized pills. Lemonade is given for thirst.

**Cinquefoil in Peritonitis.**

In the Charleston *Medical Journal*, Dr. Wm. Hauser strongly recommends cinquefoil, *Potentilla reptaus*, in puerperal peritonitis. He remarks:

"In a large practice of more than twenty years, I have never found anything, nor all other things combined, to equal this simple plant in the treatment of this exceedingly painful, dangerous and sometimes stubborn disease. I have never failed with it once, in all this time, to the best of my recollection. My method with it is simply this: Make as strong a decoction of the plant, leaves, vines and roots, as possible, and give the patient (at any stage of the case), large draughts of the tea, as hot as she can drink it, every half hour or oftener, till she be thrown into full perspiration. All pain and fever will soon be gone, and then you have the entire mastery of the case."

**Syrup of the Hypophosphites of Iron, Quinia and Strychinia.**

℞.

Freshly precipitated ferric hypophosphite, grs. 320.

Quinia (pure alkaloid) grs. 64.

Strychnia, grs. ii.

Sol. hypophosphorous acid, ℥ iss.

Syrup, sufficient to make ℥ viij. M.

Dose. Teaspoonful thrice daily.

**Gonorrhœa.**

Dr. Haberkorn, of Berlin, reports excellent results in both acute and chronic gonorrhœa, with the following injection:

℞.

Quiniæ sulphatis, gr. vj.

Glycerinæ, ʒ ij.

Aquæ, ʒ vj.

Acid, sulph. dilut., gtt. v. M.

Sig: Use a teaspoonful or two as an injection, three times a day.

**Veratrum in Laryngitis.**

In a severe case of laryngitis with laryngeal catarrh and threating apnœa, Dr. C. Handfield Jones prescribed:

℞.

Tinct. verat. virid, gtt. iv.

Aquæ, ʒ ij.

every hour until seven doses were taken; then another after two hours, and another nine hours later. Very considerable relief was promptly experienced, and recovery ensued.

**Iced Clysters in Dysentery.**

Dr. Wenzel, having had occasion to treat a great number of cases of dysentery, has found the best remedy to consist in the injection of ice-water into the rectum. It is an inoffensive, economical treatment, and gives extremely satisfactory results. The first case the doctor treated in this manner was one of severe dysentery. There were intense fever, abdominal pains, excruciating tenesmus, and profuse sanguineous evacuations. To check the hemorrhage, injections of ice-water were ordered every two hours, which not only caused the sanguineous evacuations to cease, but also removed the tenesmus, enteric pains and fever. The beneficial effects of these injections were so evident that the patient urgently demanded their repetition whenever the pains threatened to reappear. Dr. Wenzel now considers this treatment more satisfactory than any other in acute cases, although in chronic cases it can only be expected to afford a temporary and palliative effect.—*L'Independente*, April, 1874—*New York Medical Jour.*

**Neuralgia of the Larynx.**

The severe pain characterizing this affection is felt along the thyroid cartilage and anterior part of the neck. Graves recommends large doses of carbonate of iron. Gibbs has found bromide of ammonium in full doses quite successful. Dr. Clinton Wagner prefers large doses of tincture of iron and quinine, and insufflations of tanic acid and morphia into the larynx, with aconite liniment over the seat of pain.

**Cure for Burns.**

Comte de la Tour du Pui says, that a pretty strong solution of ammonia in water is an excellent remedy for burns in cases where the skin is not destroyed. Having by accident taken hold of a crucible which was nearly red hot, he plunged his hand into some ammoniacal water, and kept it some hours afterwards covered with a piece of linen soaked in the same; the pain was allayed almost immediately, and no blisters or suppuration occcurred.

**Diphtheria.**

Dr. Meyer, in the *Jahrbuch fur Kinder Krankheiten*, urges the use of ice in the treatment of this affection. In children even, under one year of age, he directs small pieces of ice to be put frequently into the mouth, followed, if possible, every minute or two, by a teaspoonful of iced water. The ice must be pure, and therefore that artificially prepared is best. In severe cases the external use of cold, by means of an ice-bag, applied around the throat, is very useful. The author has found by this mode of treatment the fever soon diminishes, and the diphtheritic membrane is detached and expectorated.

**Chloral in Sea-sickness.**

M. Giraldes, who has had very frequently to cross the Channel, and always has suffered from sea-sickness whenever the sea has been at all rough, states that some recent trials he has made of chloral have proved perfectly successful in preventing this horrid malady, even when the sea has been most tempestuous, and all his fellow-travelers have been suffering in consequence. He remains on deck, sitting quietly, and on one occasion he slept the whole passage during a furious sea. The formula he has found most useful consists of chloral three grammes (forty-five grains), distilled water, currant syrup, of each sixty grammes, and two drops of essence of mint. Of this he took one-half immediately on coming on board, and arrived at Dover without any suffering. On the return voyage he took the rest with like effect.—*Union Medicale.*

**Hysterical Spasms.**

Dr. E. Chenery, of Boston, says, in the *Medical and Surgical Journal* of that city: For many years the writer's panacea in the treatment of hysterical spasms has been the internal use of a mixture of chloroform and laudanum, after the paroxysms have been relaxed by the inhalation of chloroform, so that the patient could swallow. One good dose of this mixture has usually sufficed to explode the fits, and persons who have been frightfully convulsed have in this way been made to take up their bed and walk."

---

**Pneumonia and Bronchitis.**

A physician writing to the Boston *Journal of Chemistry*, recommends the following formula as having been very successful in his hands for 20 years:

℞.

Tinct. opii, ʒj.

Spt. etheris nitrici, ℥ss.

Syr. ipecac., ʒij.

Ol. gaultheriæ, gtt. v.

Syr. simp., ℥ijss.

M. Dose, teaspoonful every four hours for adult, or as case may require.

---

**Polk's Syrup of Ferrous Hypophosphite Comp.**

℞.

Fresh ferrous phosphate, ʒ iv.

Hyphophosphite of manganese, ʒ i.

Quinia, ʒ iss.

Strychinia, grs. iss.

Hypophosphorous acid, ʒ x.

Syrup orange flowers, sufficient to make ℥ viij. M.

Dose. A teaspoonful in water.

A powerful tonic, yet a very unstable preparation.

---

**Dilate or Cut.**

The question whether strictures of the urethra should be dilated or cut is a very practical one to surgeon and patient. At a meeting of the Medical Society of London, on the Monday before Christmas, Mr. Tuvan read a most valuable and convincing paper on the subject, which he summed up by quoting the following expression of Delpech, delivered many years ago: "Dilate when you can, cut when can't." There was a unanimous expression of opinion on the part of the Fellows present that forcible rupture of strictures was a dangerous procedure, and Mr. Tuvan mentioned that no less than thirty-nine deaths directly caused by forcible dilation had come under his notice of late years.

**Preservation and Use of Vaccine Lymph.**

Dr. Preston spreads the lymph on a morsel of paper, by means of a camel's hair pencil charged from the vaccine vesicle; he then allows it to dry. If the vaccine matter is to be kept a long time, the paper should be covered with a light coating of albumen. In order to make use of this prepared paper it is only necessary to moisten it slightly with the breath, and then apply a fragment to the abraded skin.—*New Remedies.*

---

**Ulceration of the Nose in Scrofulous Children.**

M. Galezinsky is accustomed to treat ulcerations of the cutaneous surface generally in these cases by dusting them with calomel, with appropriate internal treatment. When similar ulcerations form in the nares, he recommends similar applications, or occasionally the following ointment:

℞.

Hydrarg. ox. rub., gr. iv.
Camphoræ pulv., gr. iss.
Axungia, ℥i. M.

---

**Bromide of Ammonium in Rheumatism.**

Dr. J. M. DaCosta, Visiting Physician to the Pennsylvania Hospital, Philadelphia, while discussing rheumatism in a clinical discourse, says of this medicine, that the results he has obtained from it have been very satisfactory. He has now had the remedy on trial for more than a year, and has come to favor its use from the fact that it relieves the pain, acts generally upon the skin, keeps up the action of the kidneys, and, as he thinks, lessens materially the tendency to internal inflammations. He is further pleased with the remedy, for the reason that the cases which he has treated with it have seemed to have a shorter duration than cases treated according to other plans, and quite as short as those treated according to the ordinary alkaline plan. The advantage which it possesses over the alkaline treatment is that the recoveries are good recoveries; there is no pallor and anæmia, general distress, alteration of blood and tissues, which the long-continued administration of alkalies will cause. For these reasons he is in the habit of using the bromide of ammonium in the treatment of this class of cases. It is sometimes associated with acetate of potash, when internal complications render it desirable to have the influence of a somewhat more active diuretic.

As soon as the acute symptoms have disappeared, the remedy is followed by the administration of quinine in fair doses, which has, he believes, the power to lessen the tendency to relapses.

**Vegetable Germs the Cause of Hay-fever.**

A valuable contribution to the discussion on the germ theory of disease is made by Professor Binz, of Bonn. On investigating into the nature and true cause of the hay-fever, he discovered vegetable organisms in the nasal secretions, which were never present save during an attack of the disease. By using a neutral solution of sulphate of quinine, applied with a nasal *douche*, the animalcula were completely destroyed. In addition to the scientific value of this fact, the simplicity of the remedy will commend it to those who are afflicted with this annoying complaint.

---

**Sycosis.**

For this annoying affection Dr. Touraine, of the French military service, recommends rubbing into the eruption, morning and evening, for two days, a portion of mercurial ointment, the size of a haricot bean. The friction should be made with the finger, and should be continued until the ointment has all disappeared. The whole surface is then covered with a piece of wadding. On the third and fourth days tincture of iodine is applied, night and morning, over the mercurial friction, by means of a pencil, nothing being cleansed off. We have only to wait until the *iodine disappears*, and the cure will be complete.

---

**Treatment of Inverted Toe-Nails.**

Every practitioner, probably, is acquainted with this affection. Every one knows, also, that the various plans of treatment generally pursued are very unsatisfactory. Removal of the nail, or a part of it, is now generally considered the only certain remedy. This procedure is, to say the least, barbarous. The purpose of this article is, however, not to discuss the accepted modes of treatment, but to recommend a mode of treatment that has been employed by the writer for about four years, which has been highly satisfactory. It is simply to apply the muriated tincture of iron to the nail and the surrounding ulcerated and granulated surface, once or twice a day with a camel's-hair pencil. As a general rule, to apply it once a day, at bed-time, will be sufficient. The ulcerated surface heals with astonishing rapidity, and the nail assumes its normal appearance, making a complete cure in most cases in a few weeks. Since I commenced using this remedy, I have done nothing else in such cases. Paring and cutting the corners of the nail usually do more harm than good. I need not attempt to speak of the *modus operandi* of the remedy, the object being merely to recommend a trial of it to others.—*Dr. W. Hukill, Medical & Surgical Reporter, Phila.*

**Injection of Chloral into the Trachea.**

At a recent meeting of the Societe de Biologie, M. de Bellesme suggested the introduction of chloral into the system by this means in cases where the circulatory and absorbent functions are almost in abeyance, and where, therefore, subcutaneous or intravenous injections cannot be practised with reasonable hope of success. The process simply consists in inserting the needle of a hypordermic syringe into the trachea, at a point about one finger's-breadth below the cricoid cartilage, and slowly injecting the solution, which can be used in much larger quantities than by the subcutaneous method. This method is likely to prove serviceable in tetanus, hydrophobia, and the algid stage of cholera.

---

**Neuralgia.**

Dr. J. H. Johnson, Providence, R. I., writes to the Boston *Journal of Chemistry* as follows:—"I am daily strengthened in the conviction that this disease is asthenic in character, although the sufferer may not necessarily be of a cachectic diathesis. Decay and partial loss of the teeth, with consequent difficulty in mastication, and as a result, imperfect digestion, are among the chief causes which produce a lack of vital force, diminishing nerve nutrition, and inducing the condition known to the profession as a neuralgic diathesis. In practice I daily meet with persons suffering with neuralgia, who, upon coming under prompt and vigorous tonic treatment, are speedily restored to a normal condition, the pains vanishing as the digestive organs resume tone and strength. For several years I have used the elixir phos. ferri, quin., et strych., which has never failed to produce the desired tonic result.

I now rely upon the croton chloral to give the patient relief from all neuralgic pains until the general system can be recuperated, and brought into a condition to defy this wearing, exhausting disease. The physician in dealing with it varies his treatment to meet the requirements of different constitutions."

---

**The Treatment of Acute and Chronic Bronchitis and Asthma.**

Dr. W. H. Spurgin writes to the *British Medical Journal*, that he has tried the iodide of potassium in the treatment of these maladies, in over a hundred cases, with almost invariable success; in fact, with such success that patients have expressed themselves by saying, "it has acted like a charm;" others have said that no medicine ever had any real effect upon their complaint before. Iodide of potassium has a marked effect upon the breathing, reducing the frequency of the respirations, perhaps overcoming spasms. Almost after the first dose patients have stated they have felt the medicine touch their complaint.

He usually prescribes it with carbonate of ammonia, and, when the cough is very troublesome, adds tincture of belladonna and ipecacuanha wine.

In one case of very severe broncho-pneumonia, he tried iodide of potassium, with tincture of hyoscyamus and ammonia, and the respirations were quickly and astonishingly reduced from forty in a minute to less than half that number.

He adds, in conclusion, that he has purposely given a mixture containing ammonia, belladonna, ipecacuanha wine, spirit of sulphuric ether, etc., without iodide of potassium, without finding much benefit; after which he added iodide of potassium, and found the patient relieved almost at once.

He confidently recommends iodide of potassium as the remedy in these troublesome complaints.

---

**Catarrh.**

Dr. Dobell in his work recently published, recommends the following injection, to be used with a nasal syringe:

℞.

Boracis, ʒ j.

Glycerine carbolat, ʒ ij.

Sodæ bicarb, ʒ j.

Aquæ, tepidæ, O ss. M.

Sig. To be used night and morning.

He also used a medicated snuff three times a day, to wit:

℞.
Powdered camphor,
Tannic acid,
White sugar,
Dry Welsh snuff, āā ʒ j. M.

Also a lozenge, as:—

℞.
Powdered camphor, gr. ij.
Tannic acid, grs. ss.
Hydrochlorate of morphia, gr. 1-36.
White sugar, gr. x.
Powdered gum arabic, gr. ij.

For one lozenge.

Lastly, rub behind the ears and back of the neck, two or three times daily, with camphor liniment.

---

**Washing Out the Stomach.**

Dr. C. Ewald, of Berlin, describes a method of washing out the stomach, which, on account of its great simplicity, seems likely to make the topical treatment of diseases of the stomach, especially in cases of poisoning, much more common. "A piece of ordinary india-rubber tubing, such as is used for gas-lamps, about six feet long, is used; one end is rounded with scissors, and, if necessary, two holes are cut a short distance from the end This tube possesses quite sufficient rigidity to be passed without difficulty into the stomach. To the outer end a funnel is fitted, into which can be poured either water or a solution of soda, etc., according to circumstances. If the contents of the stomach are to be removed, the outer end of the tube must be sunk to the level of the pubes, or even lower; then the patient must make a short but forcible contraction of the abdominal walls. By this means the tube is filled to its highest point with the fluid contents of the stomach, and becomes a siphon; the liquid continuing to flow until there is no more, or till the tube is stopped up. This last seldom occurs, if the tube be of a moderate calibre. Should it, however, happen, or should the abdominal pressure be insufficient to fill the tube in the first instance, or the patient be insensible, or any similar difficulty arise, it can, in general, be readily overcome by fitting a common clyster-syringe to the end of the tube, one stroke of the piston of which is generally sufficient to remove the difficulty."

---

**Bromide of Iron in Chorea.**

Dr. J. M. DaCosta of Philadelphia, recommends in the *Medical and Surgical Reporter*, this remedy as one of great virtue in the treatment of chorea. He says: "A young girl had an attack of acute rheumatism a year ago. As she was recovering, last March, she was seized with choreic symptoms; she then had a marked mitral murmur, and the chorea was very severe. Being taken to the country, where she had fresh air and a generous diet, she rapidly convalesced, with the aid of bromide of iron. Since then repeated examinations have shown the heart to be perfectiy normal. I was led, he adds, to bromide of iron almost accidentally at first; but having now used it for three or four years, my experience, from the treatment of a large number of cases, giving abundant opportunity to witness its good effects, induce me to like it better than any other one article in the treatment of chorea. It should be given in increasing doses, never commencing with less than five grains for a child, and rapidly increasing the dose to twenty. It may be given in plain syrup and water, in the form of a pill, or better, in an effervescing powder. It not only affects the chorea, but also impresses the nervous system as a sedative, quieting it, and giving the patient rest. It is also a valuable agent in treating the incontinence of urine in children. It was in a case of this kind, complicating chorea, that I first observed its value; being surprised and pleased to see that, as the symptom which led to its administration improved, the chorea also diminished, and soon disappeared. Since then I have used it almost continuously. Local chorea, or chronic muscular spasms, such as twitching the eyelids, etc., in hysterical women, are sometimes cured by this drug, after the failure of other remedies.

